# Substantia nigra iron-related alterations, white matter disruption, and functional network dysconnectivity in temporal lobe epilepsy: a multimodal neuroimaging study

**DOI:** 10.1186/s12880-026-02613-8

**Published:** 2026-07-25

**Authors:** Xu Zhao, Huiting Wu, Si Jian, Shanshan Huang, Yingying Zhou, Tingxin Yang, Yuanhao Li, Tong Tong, Zhiqiang Zhou, Suiqiang Zhu, Xiaoyan Liu, Wenzhen Zhu

**Affiliations:** 1 Department of Radiology, Tongji Hospital, Tongji Medical College, Huazhong University of Science and Technology, Wuhan, Hubei 430030 China; 2 Department of Neurology, Tongji Hospital, Tongji Medical College, Huazhong University of Science and Technology, Wuhan, Hubei 430030 China; 3 Department of Anesthesiology and Pain Medicine, Tongji Hospital, Tongji Medical College, Huazhong University of Science and Technology, Wuhan, Hubei 430030 China

**Keywords:** Substantia nigra, Temporal lobe epilepsy, Multimodal MRI, Cognition

## Abstract

**Background:**

Although the substantia nigra (SN) has been implicated in temporal lobe epilepsy (TLE) pathophysiology, clinical studies remain scarce. This study aimed to characterize alterations in SN susceptibility, white matter (WM) microstructure, and functional connectivity (FC), as well as their associations with cognitive functions in TLE patients.

**Methods:**

This study included 20 right-TLE (RTLE), 21 left-TLE (LTLE), and 23 healthy controls (HCs). Multimodal neuroimaging was used to assess the susceptibility, diffusion tensor imaging metrics, and FC of SN subregions. Group differences were examined by analysis of covariance. Exploratory correlation analyses were performed to evaluate associations between imaging metrics and cognitive performance.

**Results:**

RTLE patients showed higher susceptibility (post-hoc *P* = 0.015 vs. HCs; *P* = 0.002 vs. LTLE) and radial diffusivity (RD) (post-hoc *P* = 0.014 vs. HCs; *P* = 0.001 vs. LTLE) in the contralateral substantia nigra pars reticulata (SNr), alongside lower fractional anisotropy (FA) (post-hoc *P* = 0.009 vs. HCs; *P* = 0.006 vs. LTLE) in the contralateral substantia nigra pars compacta (SNc). LTLE patients exhibited decreased FA in the ipsilateral SNr (post-hoc *P* < 0.001 vs. HCs; *P* = 0.001 vs. RTLE) and reduced connectivity between the left SNc and the right superior parietal lobule (*P* = 0.026, cluster-level FDR corrected). Susceptibility and RD positively correlated in the contralateral SNr of RTLE cohorts (*r* = 0.564, uncorrected *P* = 0.010). Exploratory analyses suggested associations between SNr susceptibility, FA, and cognitive performance in TLE patients (all uncorrected *P* < 0.05).

**Conclusions:**

Our multimodal findings suggest subregion- and laterality-specific alterations in the SN of patients with TLE, with patterns differing between RTLE and LTLE.

**Supplementary Information:**

The online version contains supplementary material available at https://doi.org/10.1186/s12880-026-02613-8.

## Key points

1) This study revealed nigral susceptibility changes suggestive of iron-related alterations, white matter changes, and functional reorganization in TLE.

2) RTLE and LTLE showed differential patterns of hemispheric involvement.

3) Nigral susceptibility and DTI metrics may relate to cognitive performance in TLE.

**Supplementary Information:**

The online version contains supplementary material available at https://doi.org/10.1186/s12880-026-02613-8.

## Introduction

Temporal lobe epilepsy (TLE) is the most common form of refractory focal epilepsy, severely compromising quality of life through neurological and psychological impairments [1]. While existing studies have focused primarily on limbic structures, accumulating evidence suggests that pathological abnormalities extend beyond epileptogenic zones to involve remote brain regions [2], including the substantia nigra (SN). This key basal ganglia output structure—comprising the substantia nigra pars reticulata (SNr) and the substantia nigra pars compacta (SNc)—plays a critical role in modulating seizure propagation and termination across various epilepsy types [3].

The SNr, densely populated with GABAergic neurons, controls the activity of both corticothalamic and limbic networks through its inhibitory projections [4, 5]. Several experimental studies have demonstrated seizure suppression through various SNr-targeted interventions, including direct lesions, pharmacological modulation, and optogenetic inhibition [3, 4, 6], with specific efferent projection pathways differentially regulating seizure types [7]. In contrast, the SNc—enriched with dopaminergic neurons primarily associated with Parkinson’s disease pathogenesis—has received far less attention in epilepsy research [8]. In humans, neuroimaging studies have reported SN alterations in TLE, including volume loss [9], decreased dopamine precursor uptake on positron emission tomography (PET) scans [10], and altered iron levels detected by susceptibility-weighted imaging (SWI) [11]. However, these studies have largely treated the SN as a single structure or relied on single imaging modalities, leaving the subregional and multimodal profile of SN alterations less characterized.

Advanced MRI techniques now offer opportunities to address this gap. Quantitative susceptibility mapping (QSM) overcomes the limitations of conventional SWI (e.g., blooming artifacts) and enables precise measurement of tissue magnetic susceptibility [12]. As an established method for mapping brain iron content in physiological and pathological states [13], QSM has been applied to detect subcortical tissue composition abnormalities in TLE [14]. Beyond brain iron dysregulation, epilepsy may also involve alterations in white matter (WM) microstructure. Diffusion tensor imaging (DTI) is widely used to assess microstructural integrity through metrics including fractional anisotropy (FA), axial diffusivity (AD), mean diffusivity (MD), and radial diffusivity (RD) [15]. In addition, resting-state functional magnetic resonance imaging (rs-fMRI) allows noninvasive mapping of functional connectivity (FC) alterations via blood oxygen level-dependent (BOLD) signals [16], and multimodal MRI approaches combining structural and functional imaging have been increasingly applied to characterize TLE [17, 18]. Despite these advances, few studies have combined subregional SN parcellation, multimodal imaging, and laterality-stratified comparison within a single cohort, and the integrated subregional and side-related profile of SN alterations in TLE remains less characterized. Furthermore, given the established links between these imaging metrics and cognitive performance [13, 19, 20], a comprehensive investigation is warranted.

Adopting a multimodal neuroimaging approach, this study aimed to characterize nigral susceptibility, WM microstructure, and FC in patients with TLE, and to examine side-related SN alterations between right TLE (RTLE) and left TLE (LTLE).

## Materials and methods

### Participants

**Fig. 1:**
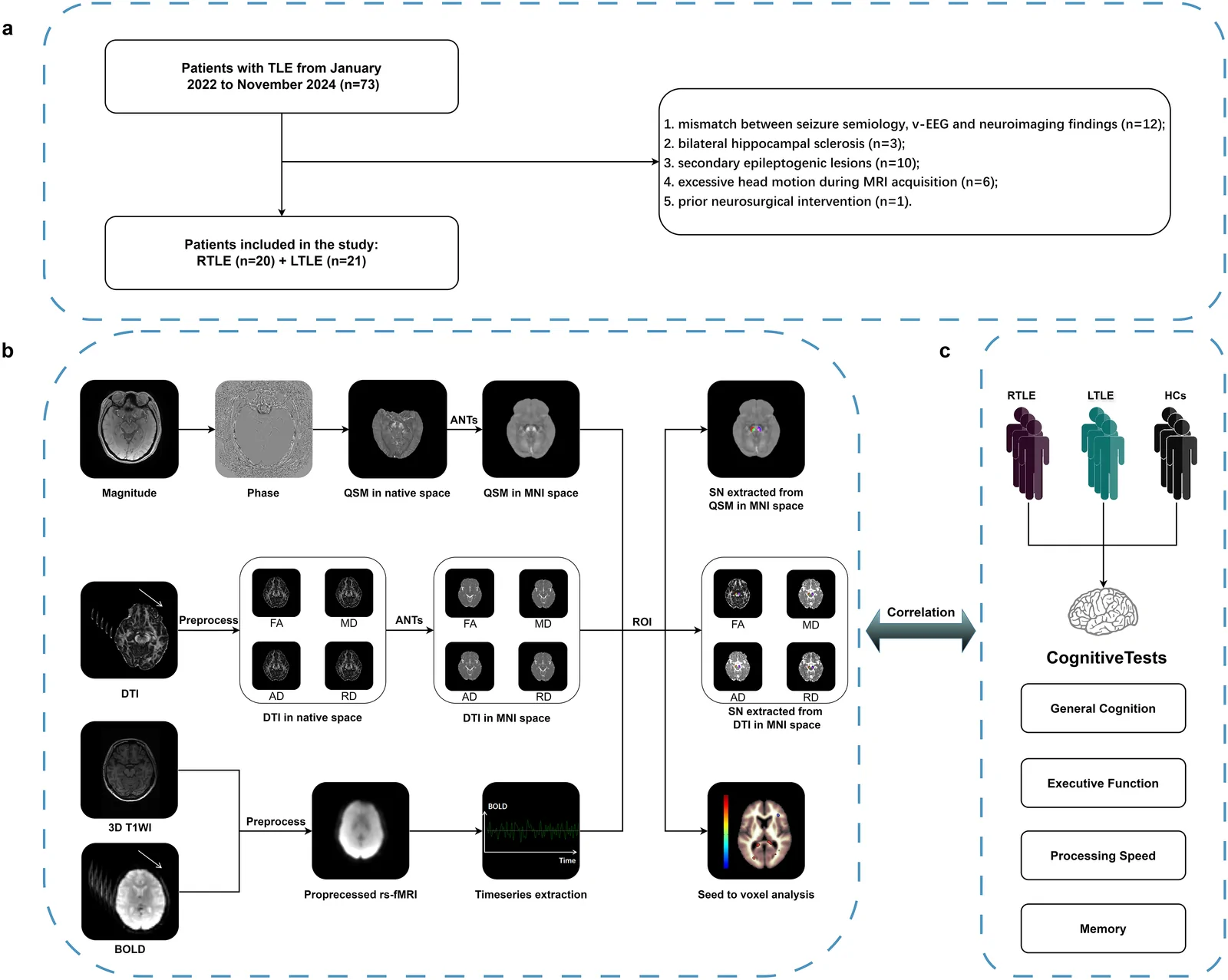
Flowchart of the study. **(a)** Patient recruitment; **(b)** Main processing steps of QSM, DTI, and rs-fMRI; **(c)** Clinical assessments. TLE, temporal lobe epilepsy; RTLE, right temporal lobe epilepsy; LTLE, left temporal lobe epilepsy; QSM, quantitative susceptibility mapping; MNI, Montreal Neurological Institute; DTI, diffusion tensor imaging; FA, fractional anisotropy; MD, mean diffusivity; AD, axial diffusivity; RD, radial diffusivity; BOLD, blood oxygen level dependent; rs-fMRI, resting-state functional magnetic resonance imaging; ROI, region of interest; HCs, healthy controls

This cross-sectional study was approved by the local institutional review board (TJ-IRB20211229) in accordance with the Declaration of Helsinki. All participants provided written informed consent. From January 2022 to November 2024, we consecutively recruited 73 participants diagnosed with TLE according to the criteria proposed by International League Against Epilepsy (ILAE) [21]. Lateralization (RTLE or LTLE) was determined through comprehensive evaluation including semiological features, video electroencephalography (v-EEG), structural neuroimaging, and/or PET. The inclusion criteria included: (1) age ≥ 18 years; (2) consent for neuroimaging; (3) right-handed; (4) no other neurological, psychiatric, or severe systemic disorders. Exclusion criteria were as follows: (1) mismatch between seizure semiology, v-EEG and neuroimaging; (2) epileptogenic lesions on MRI other than ipsilateral hippocampal sclerosis (HS) (HS was diagnosed according to typical MRI findings, including ipsilateral hippocampal atrophy, disturbed internal structure, increased signal intensity on T2-FLAIR, and widening of ipsilateral temporal angle); (3) prior neurosurgery; and (4) excessive motion artifacts on MRI. Age- and sex-matched healthy controls (HCs) were enrolled concurrently. During screening, 32 patients were excluded (Fig. 1a): 12 due to discordance among seizure semiology, v-EEG and neuroimaging findings, 3 with bilateral HS, 10 with secondary epileptogenic lesions (including 7 with malformations of cortical development and 3 with post-traumatic gliotic foci), 6 for excessive head motion during MRI acquisition, and 1 for prior epilepsy surgery. The final cohort comprised 20 RTLE (mean age, 29.0 ± 7.3 years; male: female, 7:13), 21 LTLE (mean age, 30.4 ± 8.2 years; male: female, 11:10) and 23 HCs (mean age, 31.1 ± 7.4 years; male: female, 9:14). For each patient, we recorded age at epilepsy onset, disease duration, seizure frequency, and current antiseizure medication (ASM) regimen.

### Image acquisition

All scans were obtained using a 3.0 T MRI scanner (Discovery MR750; GE Healthcare, Milwaukee, Wisconsin) with a 32-channel head coil. Participants were instructed to remain still, with head motion minimized using conformable foam padding. Sagittal three-dimensional T1-weighted imaging brain volume (3D-T1 BRAVO) used the following parameters: TR/TE/TI 8.2/3.2/450 ms, flip angle 12°, slice thickness 1 mm, sagittal slices 166, matrix size 256 × 256 × 160, FOV 25.6 × 25.6cm^2^, and NEX = 1. DTI was acquired with: TR/TE 8500/66.3 ms, FOV 25.6 × 25.6 cm^2^, matrix size 128 × 128, slice thickness 2 mm, number of slices 70, number of diffusion gradient directions 64, *b* = 0 and 1000 s/mm^2^, number of images at a b-value of 0 s/mm^2^ = 5, acceleration factor 2, and scan time 9 min 55 s. The parameters of 3D multi-gradient echo (mGRE) were as follows: TR = 42.8 ms, TE1/ΔTE = 4.5/4.9 ms, number of TEs = 8, matrix = 416 × 320, readout bandwidth = 244 Hz/pixel, slice thickness = 2 mm, flip angle = 20°, FOV = 240 × 240 mm^2^. The rs-fMRI scans were obtained using a Gradient-recalled echo-planar imaging (GRE-EPI) sequences with the following parameters: TR/TE 2000/35 ms, flip angle = 90°, FOV = 240 × 240 mm^2^, matrix = 64 × 64, axial slices = 40, slice thickness = 4 mm with no gap, scan time = 8 min, 240 volumes for each subject.

### Multimodal images processing

The data processing flow is presented in Fig. 1b.

#### QSM processing

The QSM reconstruction was performed using the morphology-enabled dipole inversion with an automatic uniform cerebrospinal fluid (CSF) zero-reference algorithm (MEDI + 0) on mGRE datasets [22]. First, the total field estimation was achieved through a nonlinear fitting of the mGRE data [23]. Subsequently, the projection onto dipole fields (PDF) algorithm [24] was implemented for effective background field removal, generating the computation of the local field. To enhance image quality, the regularization enforcing uniform susceptibility distribution within CSF was applied, coupled with spatial co-registration to the magnitude images. The ventricular CSF mask was achieved using R2^*^ maps derived from the mGRE magnitude data through threshold segmentation and connectivity analysis. Finally, the native QSM maps were registered to the QSM template space [25] using ANTsSyN script (ANTs version 2.3.0).

#### DTI processing

The DTI data were preprocessed using FSL tools (http://www.fmrib.ox.ac.uk/fsl). First, the raw DICOM images were converted to NIFTI format. Then, eddy current-induced distortions and motion artifacts were subsequently corrected using the ‘eddy-correct’ function of FSL. Following stripping non-brain tissues using the Brain Extraction Tool (BET), diffusion tensor maps were reconstructed via DTIFIT, from which quantitative diffusion metrics were derived. The native FA, MD, AD, and RD maps were registered to the standard Montreal Neurological Institute (MNI) space using ANTsSyN script (ANTs version 2.3.0).

#### Rs-fMRI processing

Functional images were preprocessed using CONN toolbox (v17; Whitfield-Gabrieli and Nieto-Castanon, 2012; www.nitrc.org/projects/conn) implemented in MATLAB (R2017a; MathWorks, Natick, MA). Preprocessing steps included: removal of the first 10 volumes to ensure steady-state magnetization, realignment, slice-time correction, normalization, outlier detection (ART-based scrubbing method [26]), spatial smoothing and temporal filtering, and structural segmentation/normalization to the MNI space. To remove unwanted motion, physiological and other artifactual effects from the BOLD signal, a comprehensive protocol was applied, incorporating: component-based noise correction (aCompCor) (WM and cerebrospinal fluid, five components each), scrubbing, motion regression (default 12 regressors), and filtering. Further, FC analysis between the bilateral SN subregions and the whole brain was performed with a seed-to-voxel approach. Pearson’s correlation coefficients quantified FC strength between BOLD signals in the seed regions and other brain voxels. These coefficients were then converted into normally distributed scores using Fisher’s Z-transformation for general linear model analyses.

### ROI-based analysis

Bilateral SNr and SNc were defined as ROIs based on a whole-brain QSM parcellation map [25] that integrates the AAL2 atlas, the JHU DTI-based WM atlas and a deep gray matter nuclei parcellation. All ROI-based parameter extractions were performed in standardized space: the native QSM maps were registered to the QSM template space, and the DTI and rs-fMRI images were normalized to MNI space during preprocessing. The atlas-derived parcellation map, which is defined in the QSM template/MNI space, was then applied to these normalized images to extract mean parameter values and to define seed regions for the FC analysis.

To ensure accurate ROI placement, all individual registration results—including QSM-to-template, DTI-to-MNI, and rs-fMRI-to-MNI alignments—were visually inspected by SJ and TY. Specifically, we checked the alignment of key midbrain anatomical landmarks (the red nucleus, the SN, and the interpeduncular fossa) in the axial, coronal, and sagittal planes. Any participant with a visible misalignment > 2 mm in any plane, or with obvious failure of the non-linear warp affecting the midbrain or brainstem, underwent manual ROI correction and re-registration.

For all analyses, ‘ipsilateral’ was defined as the SN subregion on the same side as the epileptogenic focus (i.e., the seizure-onset temporal lobe), and ‘contralateral’ as the opposite side. Specifically, for RTLE patients, the right side was considered ipsilateral and the left side contralateral; for LTLE patients, the left side was considered ipsilateral and the right side contralateral. This definition was applied consistently across all imaging metrics and correlation analyses. Mean parameter values were extracted from the ipsilateral and contralateral SN subregions for each patient; for the HC group, the average of both hemispheres was used.

### Neuropsychological assessments

Patients underwent neuropsychological testing (Fig. 1c; detailed in Supplementary Materials and Methods), including: Montreal Cognitive Assessment (MoCA), Digit Symbol Substitution Test (DSST), Digit Span Test (DST), Verbal Fluency Test (VFT), and Auditory Verbal Learning Test-HuaShan version (AVLT-H). One patient was missing MoCA scores, and nine were missing scores for DSST, DST, VFT, and AVLT-H.

### Statistical analysis

Demographic and clinical data were analyzed using SPSS (version 26; IBM Corp., Armonk, NY, USA). Normally distributed quantitative variables were expressed as mean ± standard deviation and compared by one-way analysis of variance (ANOVA). Non-normally distributed continuous variables were reported as medians (interquartile ranges), compared using the Kruskal-Wallis test. Categorical variables were presented as frequencies (%), compared by the chi-square test or Fisher’s exact test.

For between-group comparisons of regional imaging metrics, analysis of covariance (ANCOVA) was performed with age and sex as covariates. To control for multiple comparisons across the four SN subregions, Bonferroni correction was applied, with statistical significance set at *P* < 0.0125. Post hoc pairwise comparisons used LSD adjustment.

To directly assess whether laterality patterns differed between RTLE and LTLE, we performed post-hoc 2 (Group: RTLE vs. LTLE) × 2 (Side: ipsilateral vs. contralateral) mixed ANCOVAs for each imaging metric, with age and sex as covariates. These analyses were conducted only in the two patient groups, as ‘ipsilateral’ and ‘contralateral’ are defined relative to the epileptogenic focus and lack a biological correlate in HCs.

For exploratory whole-brain seed-to-voxel FC analyses, cluster-level false discovery rate (FDR) correction was applied (q < 0.05) at the cluster level following a voxel-wise threshold of *P* < 0.001. For exploratory imaging-cognition correlations, Spearman correlation analyses were performed separately for each imaging modality. Within each modality, FDR correction was applied to the set of all correlation tests (q < 0.05). Correlations with uncorrected *P* < 0.05 but q > 0.05 are reported as exploratory and interpreted with caution.

## Results

### Demographic and clinical characteristics

A total of 20 RTLE patients, 21 LTLE patients, and 23 HCs (all right-handed) were included (Table 1), with no significant between-group differences in age (*P* = 0.671) or sex distribution (*P* = 0.495). RTLE and LTLE groups did not differ significantly in age of onset, disease duration, seizure frequency, or neuropsychological performance (all *P* > 0.05). The prevalence of HS was significantly higher in the RTLE group compared with the LTLE group (*P* = 0.019). The majority of patients in both groups were on polytherapy, with no intergroup difference (*P* = 0.943). The most commonly prescribed medications were valproic acid and lamotrigine in both groups, and the proportion of patients receiving each specific ASM did not differ between groups (all *P* > 0.05).

**Table 1:** Demographic and clinical characteristics of the participants

Variables	HC (*n* = 23)	RTLE (*n* = 20)	LTLE (*n* = 21)	*P* ^*^
Age, yr	31.1 ± 7.4	29.0 ± 7.3	30.4 ± 8.2	0.671
Sex, male: female	9:14	7:13	11:10	0.495
Age onset, yr	-	20.4 ± 7.8	18.1 ± 7.3	0.345
Duration, yr	-	8.7 ± 6.6	13.0 ± 9.0	0.092
Seizure frequency, n (%)				0.269
≥ 1/month	-	18 (90.0)	15 (71.4)	
< 1/month	-	2 (10.0)	6 (28.6)	
Drug-resistant epilepsy, n (%)	-	14 (70.0%)	15 (71.4%)	0.920
Hippocampal sclerosis, n (%)	-	13 (65.0)	6 (28.6)	0.019
Treatment types, n (%)				0.943
Monotherapy	-	4 (20.0)	3 (14.3)	
Polytherapy	-	16 (80.0)	18 (85.7)	
Individual ASM prescribed, n (%)				
Valproic acid	-	11 (55.0)	12 (57.1)	0.890
Lamotrigine	-	11 (55.0)	12 (57.1)	0.890
Oxcarbazepine	-	4 (20.0)	7 (33.3)	0.335
Topiramate	-	5 (25.0)	5 (23.8)	>0.999
Levetiracetam	-	4 (20.0)	4 (19.0)	>0.999
Lacosamide	-	2 (10.0)	3 (14.3)	>0.999
Carbamazepine	-	2 (10.0)	2 (9.5)	>0.999
Perampanel	-	1 (5.0)	2 (9.5)	>0.999
MoCA, score (*n* = 40)	-	24.8 ± 3.7	24.8 ± 3.7	0.987
DSST, score (*n* = 32)	-	57.4 ± 11.2	61.8 ± 16.2	0.378
DST, score (*n* = 32)	-	13.1 ± 3.5	12.9 ± 2.8	0.909
VFT, score (*n* = 32)	-	15.0 ± 5.2	12.3 ± 5.3	0.165
AVLT-H, score (*n* = 32)				
Short-delayed recall	-	8.6 ± 2.0	7.4 ± 1.9	0.075
Long-delayed recall	-	8.6 ± 1.7	8.1 ± 2.0	0.444

Variables are presented as the frequencies (%) or mean ± standard deviation

^*^P-values less than 0.05 were considered significant

HC, healthy control; RTLE, right temporal lobe epilepsy; LTLE, left temporal lobe epilepsy; ASM, antiseizure medication; MoCA, Montreal Cognitive Assessment; DSST, Digit Symbol Substitution Test; DST, Digit Span Test; VFT, Verbal Fluency Test; AVLT-H, Auditory Verbal Learning Test-HuaShan version

### Group differences in imaging metrics

#### Susceptibility

**Fig. 2:**
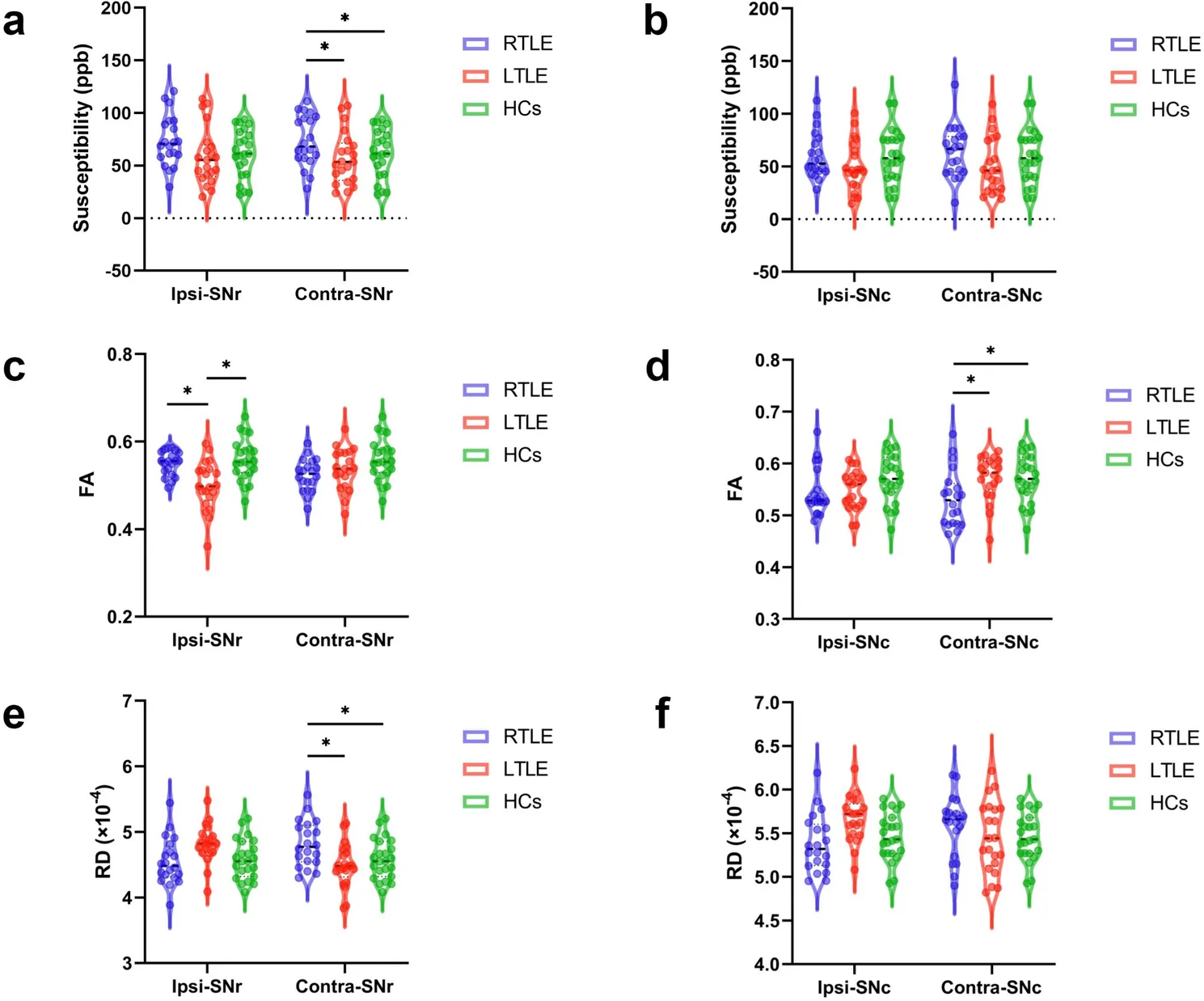
Comparisons of susceptibility and DTI metrics in substantia nigra subregions among RTLE, LTLE, and HCs. **(a)** Susceptibility in SNr. **(b)** Susceptibility in SNc. **(c)** FA in SNr. **(d)** FA in SNc. **(e)** RD in SNr. **(f)** RD in SNc. ^*^P < 0.0125 was considered statistically significant. For HCs, values represent the average of bilateral measurements. Violin plots show the full data distribution; points represent individual participant values; internal lines indicate the median. DTI, diffusion tensor imaging; RTLE, right temporal lobe epilepsy; LTLE, left temporal lobe epilepsy; HCs, healthy controls; SNr, substantia nigra pars reticulata; SNc, substantia nigra pars compacta; FA, fractional anisotropy; RD, radial diffusivity; Ipsi, ipsilateral to epileptic focus; Contra, contralateral to epileptic focus. For healthy controls, mean parameter values from both hemispheres were used for comparison with patient ROIs ipsilateral or contralateral to the epileptogenic foci

‘Ipsilateral’ and ‘contralateral’ were defined relative to the side of the epileptogenic focus (see Methods). ANCOVA revealed a significant group effect for susceptibility in the contralateral SNr (F(2, 58) = 5.548, *P* = 0.006, η²*p* = 0.161), which withstood Bonferroni correction (*P* < 0.0125). Post-hoc pairwise comparisons showed that RTLE patients exhibited significantly higher susceptibility in the contralateral SNr compared to both HCs (mean difference = 15.891, *P* = 0.015, Cohen’s d = 0.780, 95% CI [3.192, 28.590]) and LTLE (mean difference = 20.535, *P* = 0.002, Cohen’s d = 1.008, 95% CI [7.620, 33.450]; Fig. 2a). No group differences were observed in the SNc (*P* = 0.082; Fig. 2b).

#### Diffusion data

ANCOVA revealed significant group effects for FA in the ipsilateral SNr (F(2, 58) = 10.065, *P* < 0.001, η²*p* = 0.258), FA in the contralateral SNc (F(2, 58) = 5.04, *P* = 0.010, η²*p* = 0.148), and RD in the contralateral SNr (F(2, 58) = 6.847, *P* = 0.002, η²*p* = 0.191), all surviving Bonferroni correction (*P* < 0.0125). Post-hoc pairwise comparisons showed that the RTLE group had significantly lower FA in the contralateral SNc than both HCs (mean difference = − 0.040, *P* = 0.009, Cohen’s d = 0.894, 95% CI [–0.070, − 0.010]) and LTLE patients (mean difference = − 0.043, *P* = 0.006, Cohen’s d = 0.961, 95% CI [–0.074, − 0.013]), and higher RD in the contralateral SNr than both HCs (mean difference = 0.249, *P* = 0.014, Cohen’s d = 0.79, 95% CI [0.052, 0.445]) and LTLE patients (mean difference = 0.362, *P* = 0.001, Cohen’s d = 1.15, 95% CI [0.162, 0.562]). For LTLE patients, post-hoc comparisons revealed significantly lower FA in the ipsilateral SNr than both HCs (mean difference = − 0.059, *P* < 0.001, Cohen’s d = 1.32, 95% CI [–0.086, − 0.031]) and RTLE patients (mean difference = − 0.049, *P* = 0.001, Cohen’s d = 1.10, 95% CI [–0.077, − 0.020]). These results are detailed in Fig. 2c-f. No significant intergroup differences in AD or MD values were detected (all *P* > 0.05; Supplementary Fig. 1).

#### Interaction analysis of laterality effects

To formally assess whether laterality patterns differed between RTLE and LTLE, we performed 2 (Group: RTLE vs. LTLE) × 2 (Side: ipsilateral vs. contralateral) mixed ANCOVAs with age and sex as covariates for each imaging metric. The results are summarized in Supplementary Table 1. For DTI metrics, significant Group × Side interactions were observed for FA and RD in both the SNr and SNc, as well as for MD in the SNr (all *P* < 0.05). Simple effects analyses showed that in the LTLE group, the ipsilateral SNr exhibited significantly lower FA (*P* < 0.001), higher RD (*P* < 0.001), and higher MD (*P* = 0.010) compared with the contralateral side, and the ipsilateral SNc exhibited significantly lower FA (*P* = 0.005) and higher RD (*P* = 0.008). In the RTLE group, the contralateral SNr exhibited significantly lower FA (*P* = 0.003) and higher RD (*P* = 0.003) compared with the ipsilateral side, and the contralateral SNc exhibited significantly higher RD (*P* < 0.001). For QSM susceptibility, the Group × Side interaction did not reach significance in either the SNr or SNc (both *P* > 0.05). To assess the potential influence of HS on the observed laterality patterns, we repeated the interaction analyses including HS as an additional covariate. The results remained essentially unchanged (Supplementary Table 2).

### Exploratory analyses

#### Functional connectivity

**Fig. 3:**
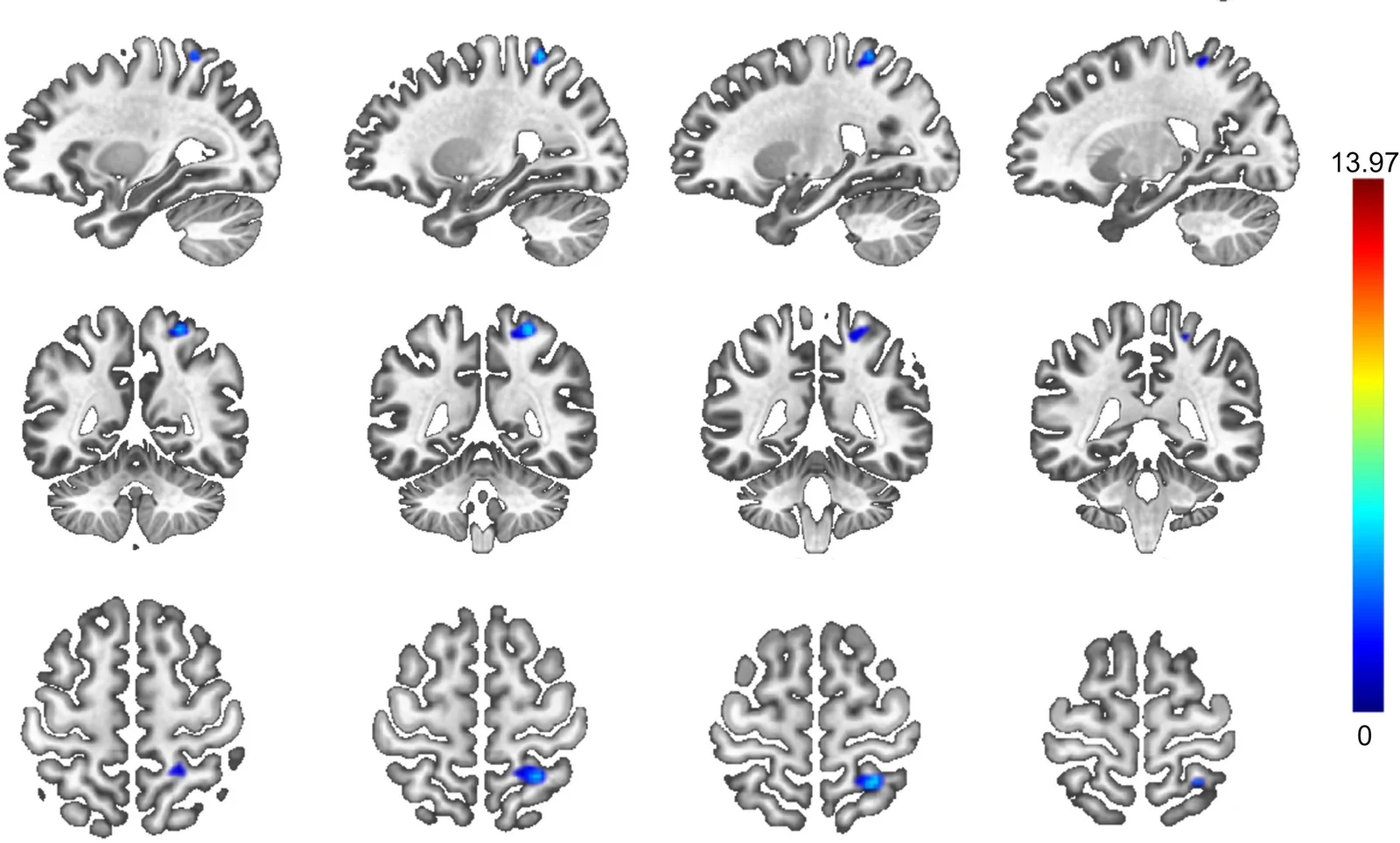
Decreased left SNc-right SPL connectivity in LTLE. Brain maps showing reduced functional connectivity between the left SNc and the right SPL in patients with LTLE compared with HCs. SNc, substantia nigra pars compacta; SPL, superior parietal lobule; LTLE, left temporal lobe epilepsy; HCs, healthy controls

FC analysis revealed reduced connectivity between the left SNc and right superior parietal lobule (SPL) in LTLE patients versus HCs (mean difference = − 0.171, 95% CI [–0.242, − 0.100], Cohen’s d = 1.47, *P* = 0.026, cluster-level FDR corrected; Fig. 3). No significant FC alterations were observed in RTLE group when using SNr or SNc as seed regions (all *P* > 0.05).

#### Imaging-cognition correlations

**Fig. 4:**
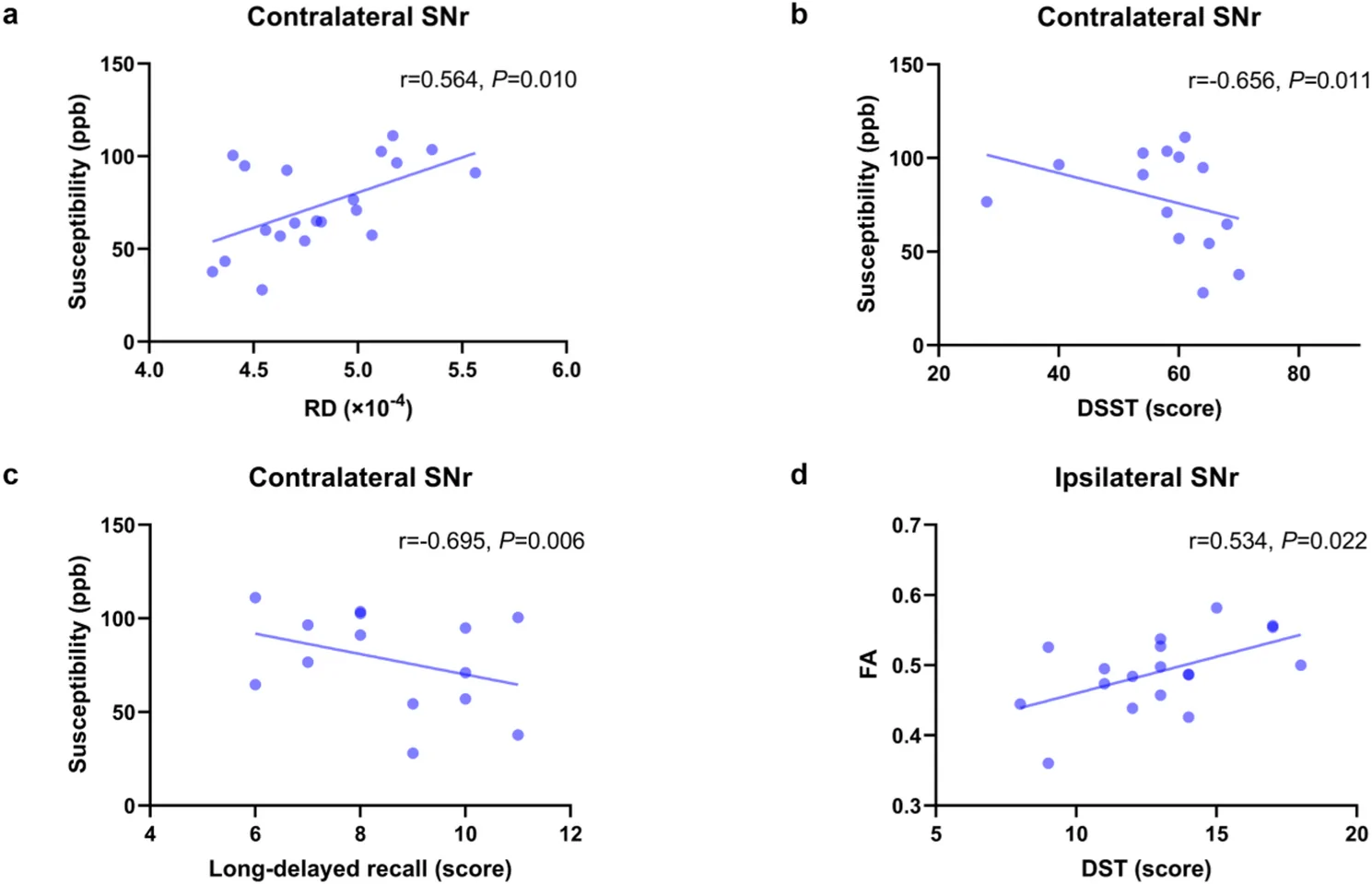
Correlations of MRI metrics with cognitive performance in temporal lobe epilepsy. **(a)** Susceptibility in the contralateral SNr positively correlated with RD in RTLE (*n* = 20). **(b)** Susceptibility in the contralateral SNr negatively correlated with DSST scores in RTLE (*n* = 14). **(c)** Susceptibility in the contralateral SNr negatively correlated with long-delayed recall scores in RTLE (*n* = 14). **(d)** FA in the ipsilateral SNr positively correlated with DST scores in LTLE (*n* = 18). All *P*-values shown are uncorrected; none remained significant after FDR correction. SNr, substantia nigra pars reticulata; RD, radial diffusivity; DSST, Digit Symbol Substitution Test; DST, Digit Span Test; FA, fractional anisotropy; LTLE, left temporal lobe epilepsy; RTLE, right temporal lobe epilepsy

In patients with RTLE, convergent microstructural alterations were localized exclusively to the contralateral SNr. Within this region, susceptibility showed a significant positive correlation with RD (*n* = 20; *r* = 0.564, uncorrected *P* = 0.010; Fig. 4a), with no cross-modal spatial concordance observed elsewhere. Moreover, susceptibility in the contralateral SNr correlated negatively with DSST scores (*n* = 14; *r* = -0.656, uncorrected *P* = 0.011; Fig. 4b) and with long-delayed recall performance (*n* = 14; *r* = -0.695, uncorrected *P* = 0.006; Fig. 4c). In LTLE patients, lower FA in the ipsilateral SNr was associated with reduced DST scores (*n* = 18; *r* = 0.534, uncorrected *P* = 0.022; Fig. 4d). However, none of these correlations survived FDR correction for multiple comparisons (all q > 0.05). No significant correlations were observed between the FC of the left SNc-right SPL and any measures of cognitive performance (all uncorrected *P* > 0.05).

## Discussion

Our multimodal neuroimaging study extends prior work by characterizing microstructural and functional alterations of the SN in TLE. The main findings are as follows: (1) RTLE patients exhibited elevated susceptibility and increased RD in the contralateral SNr, alongside decreased FA in the contralateral SNc. Within the contralateral SNr, susceptibility was positively correlated with RD. (2) LTLE patients showed reduced FA in the ipsilateral SNr, along with reduced FC between the left SNc and the right SPL. (3) Interaction analyses showed that DTI-based laterality patterns—particularly FA and RD—were statistically distinguishable between RTLE and LTLE, with ipsilateral-predominant involvement in LTLE and contralateral-predominant involvement in RTLE; for QSM susceptibility, however, the interaction did not reach significance. These alterations exhibited hemispheric-lateralized patterns that corresponded to the side of the epileptic focus. (4) Exploratory analyses revealed preliminary associations between nigral alterations and cognitive performance. Taken together, these results suggest that the SN may be differentially involved in RTLE and LTLE, with laterality patterns primarily observed in DTI metrics. However, these findings are preliminary and should be interpreted with caution, as the modest sample size limits both the robustness of the observed effects and the statistical power to detect smaller differences.

Recent optogenetic studies have demonstrated the SNr’s role in seizure modulation. Wicker et al. [7] demonstrated that silencing specific efferent pathways—SNr-to-superior colliculus and SNr-to-pedunculopontine nucleus projections—differentially controls seizure types. Subsequently, Chen et al. [4] revealed that activating SNr GABAergic neurons exacerbates seizure activities, while inhibiting them suppresses seizures, and further identified a long-range SNr-parafascicular disinhibitory circuit regulating seizures. Although animal studies have confirmed SNr involvement in TLE, human studies characterizing nigral dysregulation are limited.

In patients with RTLE, we observed increased susceptibility specifically within the contralateral SNr, which is consistent with iron-related changes. This finding aligns with previous literature implicating iron overload in epileptogenesis, as demonstrated in post-hemorrhagic or post-traumatic rodent models [27, 28] and supported by clinical studies linking dysregulated serum iron and inflammation to seizures in focal epilepsy [29]. A recent study further reported ferritin overexpression and iron retention in brain tissue from patients with status epilepticus and TLE [30], likely resulting from blood-brain barrier compromise and altered intracellular iron management facilitated by paroxysmal seizure activity. While previous SWI research reported decreased whole-SN susceptibility in mesial TLE [11], our QSM analysis identified a focal increase in susceptibility specifically within the contralateral SNr of RTLE patients. This discrepancy likely stems from the higher sensitivity and specificity of QSM in quantifying regional susceptibility, combined with our subregion-focused analytical approach. These findings raise the possibility that recurrent seizures may promote susceptibility changes—possibly reflecting iron accumulation—even in remote subcortical structures such as the SNr. Unfortunately, we did not obtain biopsy or post-mortem brain tissue samples, which precluded histological validation of iron distribution through direct staining methods [31, 32].

Microstructurally, the RTLE cohort exhibited increased RD in the contralateral SNr and reduced FA in the contralateral SNc. Elevated RD is a sensitive marker of myelin abnormalities [33], while reduced FA typically indicates compromised microstructural integrity [34]. These findings are consistent with experimental evidence confirming loss of myelin and oligodendrocytes during epileptogenesis [35, 36], and with clinical neuroimaging studies showing widespread WM impairment in epilepsy [37, 38]. Regarding the SNc, we found decreased FA on the contralateral side of RTLE patients. Although the SNc’s role in TLE remains poorly understood, recent animal evidence has supported dopaminergic SNc involvement in epilepsy, indicating its potential as a therapeutic target for modulating epileptiform activities [39]. The underlying mechanisms warrant further investigation.

Notably, susceptibility in the contralateral SNr in RTLE patients showed a significant positive correlation with RD. This relationship is consistent with prior findings, where elevated cerebral iron is associated with reduced WM integrity [40, 41]. Mechanistically, while iron serves as an essential cofactor for enzymes involved in myelin synthesis [42], excess iron can trigger oxidative stress, neuroinflammation, and glutamate excitotoxicity, potentially contributing to WM injury [42, 43]. Therapeutic interventions for iron overload have been shown to alleviate such WM damage experimentally [44]. Conversely, WM injury may itself disrupt iron transport, creating a vicious cycle [45]. Despite recognition of these interactions, the synergistic relationship between iron deposition and WM pathology in TLE remains poorly characterized, necessitating further exploration.

In patients with LTLE, a distinct pattern of alterations emerged. Microstructural damage was characterized by reduced FA in the ipsilateral SNr, indicating compromised WM integrity on the same side as the epileptic focus. Functionally, we identified disrupted FC between the left SNc and the right SPL. As a part of the sensorimotor network, the SPL maintains body balance and coordinates movement [46, 47]. The observed network alterations may reflect dynamic reorganization that might affect distal neural function, potentially relevant to motor seizure manifestations.

The lateralized patterns observed in DTI metrics—characterized by predominantly contralateral alterations in RTLE versus ipsilateral alterations in LTLE—suggest differential hemispheric involvement between the two groups. These asymmetries align with prior neuroimaging studies that have reported greater contralateral involvement in RTLE compared with LTLE using fMRI [48], DTI [49], and morphometry [50], potentially related to interhemispheric neurodevelopmental differences [51] or distinct reorganization patterns during epileptogenesis [52]. While some studies have reported more extensive abnormalities in LTLE [53, 54], such cross-study discrepancies may stem from methodological heterogeneity and cohort differences (e.g., sample sizes, clinical profiles). Our findings also suggest that contralateral SN alterations in RTLE may reflect compensatory network reorganization, potentially related to disrupted bilateral limbic and neocortical input or impaired ventral striatum-SNr GABAergic function [55]. Whether these multimodal imaging features could ultimately contribute to lateralization in clinically challenging cases remains speculative and would require validation in dedicated prospective studies. Of note, the interaction patterns differed across modalities—QSM susceptibility did not reach significance, whereas DTI metrics showed significant Group × Side interactions. This dissociation suggests that susceptibility changes and microstructural alterations may reflect different aspects of SN pathology in TLE, with DTI showing stronger laterality specificity in this cohort. These findings highlight the complementary value of multimodal imaging in characterizing SN alterations in TLE.

Furthermore, the study suggested potential associations between alterations in the SN and cognitive function. In RTLE, susceptibility in the contralateral SNr correlated with lower DSST scores (processing speed) and poorer long-delayed recall performance (memory). In LTLE, reduced FA in the ipsilateral SNr was associated with DST performance (working memory). These links between susceptibility changes, WM microstructure, and domain-specific cognitive performance align with findings in other disorders where deep gray matter pathology contributes to cognitive deficits [13, 56, 57]. The SNr’s involvement in reward, motor, and learning circuits, as well as dorsolateral prefrontal circuits—critical for executive functioning [56]—provides a neuroanatomical basis for these observations. Taken together, these exploratory findings suggest that iron-related susceptibility changes and microstructural alterations in the SNr may be associated with cognitive performance in TLE. However, it is important to note that although several nominally significant associations were observed, none survived correction for multiple comparisons; therefore, these results should be interpreted cautiously and require validation in larger independent cohorts.

Several methodological considerations regarding the SN subregion analysis warrant discussion. First, although the native QSM resolution (0.58 × 0.75 × 2.0 mm^3^) is anisotropic and limits the precise delineation of SNc/SNr boundaries, we followed Zhang et al. [21] by resampling all images to 1 mm isotropic and employing ANTs SyN diffeomorphic registration to achieve sub-voxel alignment. Our findings therefore reflect regional rather than sub-millimeter localization. Second, to minimize partial volume effects—a particular concern for small brainstem nuclei—we utilized the QSM-based deep gray matter parcellation map from Zhang et al. [21], in which the SN was manually re-drawn directly on QSM contrast rather than on T1-weighted images, substantially reducing contamination from adjacent WM tracts. Third, the reliability of this atlas-based approach for SN subregions is supported by the quantitative validation reported in Zhang et al. [21], with overall Dice ratios > 0.84 for adult datasets when compared against manual ground-truth. Finally, all individual registrations underwent rigorous manual quality control by visual inspection of midbrain landmarks; cases with misalignment > 2 mm were subjected to manual ROI correction. These methodological strategies collectively support the robustness of our SN subregion analyses.

### Limitations

This study had several limitations. First, the small sample size limited the statistical power to detect small effects, and the cross-sectional design precluded causal inferences or tracking of disease progression. Longitudinal investigations with larger cohorts are needed. Second, the correlation analyses were exploratory in nature: although FDR correction was applied, none of the associations survived correction for multiple comparisons, and effective sample sizes varied due to incomplete neuropsychological data. These findings should therefore be interpreted as preliminary and hypothesis-generating rather than confirmatory. Third, while increased susceptibility is commonly interpreted as consistent with iron accumulation based on prior QSM studies [14, 58], other trace metals, such as copper and aluminum, may also contribute. Histopathological validation is required to confirm our neuroimaging findings. Fourth, the prevalence of HS differed significantly between RTLE and LTLE groups. We acknowledge that this between-group difference represents an important clinical context when interpreting the observed SN alterations. Whether these SN changes arise from primary nigral pathology or reflect secondary consequences of HS-related mesial temporal and limbic network dysfunction remains unclear. Supplementary analyses adjusting for HS did not substantially alter the main results; however, we cannot completely exclude the influence of HS-related pathology. Future studies with HS-matched cohorts are needed to further clarify these relationships. Fifth, ASMs may influence both MRI metrics and cognitive performance, and their potential confounding effects—such as the number of ASMs or total daily defined dose—should be systematically examined in future research with larger cohorts. Sixth, regarding image processing precision, although we implemented QSM-based ROI definition to minimize partial volume effects, adopted the validated multi-atlas pipeline of Zhang et al. [25] with ANTs SyN diffeomorphic registration, and performed rigorous manual quality control on all registrations, the native through-plane resolution (2.0 mm) still limits the precise delineation of SNc/SNr boundaries, and atlas-based registration for small brainstem nuclei remains inherently susceptible to inter-individual anatomical variability. Future studies employing isotropic high-resolution QSM (≤ 1 mm^3^) or ultra-high-field 7T MRI would further improve subregional accuracy.

## Conclusion

In summary, our findings provide preliminary evidence that subregional SN alterations in TLE may show laterality-related differences that vary by imaging modality. Given the exploratory nature of these analyses and the modest sample size, these results should be interpreted cautiously and require replication in larger independent cohorts.

## Supplementary Information

Below is the link to the electronic supplementary material.

Supplementary Material 1

## Data Availability

The data used during the current study are available from the corresponding author upon reasonable request.

## Abbreviations

3D-T1 BRAVO: Sagittal three-dimensional T1-weighted imaging brain volume
AD: Axial diffusivity
ANCOVA: analysis of covariance
ANOVA: Analysis of variance
AVLT-H: Auditory Verbal Learning Test-HuaShan versio
BET: Brain Extraction Tool
BOLD: Blood oxygen level-dependent
CSF: Cerebrospinal fluid
DTI: Diffusion tensor imaging
DSST: Digit Symbol Substitution Test
DST: Digit Span Test
FA: Fractional anisotropy
FC: Functional connectivity
GRE-EPI: Gradient-recalled echo-planar imaging
HCs: Healthy controls
ILAE: International League Against Epilepsy
LTLE: Left temporal lobe epilepsy
MD: Mean diffusivity
mGRE: Multi-gradient echo
MNI: Montreal Neurological Institute
MoCA: Montreal Cognitive Assessment
PDF: Projection onto dipole fields
QSM: Quantitative susceptibility mapping
RD: Radial diffusivity
rs-fMRI: Resting-state functional magnetic resonance imaging
RTLE: Right temporal lobe epilepsy
SN: Substantia nigra
SNc: Substantia nigra pars compacta
SNr: Substantia nigra pars reticulate
SPL: Superior parietal lobule
SWI: Susceptibility-weighted imaging
TLE: Temporal lobe epilepsy
v-EEG: Video electroencephalography
VFT: Verbal Fluency Test
WM: White matter
