# Supplementary material for: Substantia nigra iron-related alterations, white matter disruption, and functional network dysconnectivity in temporal lobe epilepsy: a multimodal neuroimaging study
Source: BMC Med Imaging. 2026 Jul 25;26:547. doi: 10.1186/s12880-026-02613-8 (PMC13700436; doi:10.1186/s12880-026-02613-8)
Supplement: Supplementary file 1 [file 12880_2026_2613_MOESM1_ESM.docx]

**SUPPLEMENT**

**Supplementary Materials and Methods**

Prior to undergoing MRI scanning, patients completed a comprehensive battery of neuropsychological tests assessing: (a) global cognition: Montreal Cognitive Assessment (MoCA); (b) processing speed: Digit Symbol Substitution Test (DSST); (c) executive function: Digit Span Test (DST) and Verbal Fluency Test (VFT); and (d) memory: Auditory Verbal Learning Test-HuaShan version (AVLT-H).

The MoCA, a widely used 30-item screening tool for cognitive impairment [1], evaluates orientation, figure drawing, processing speed, naming, memory, recall, attention, vigilance, repetition, verbal fluency, and abstraction.

The DSST is a pencil-and-paper test requiring participants to match symbols to numbers using a reference grid. Scores reflect the number of correct matches completed within 90 seconds [2].

The DST [3] presents random digit sequences. Participants verbally repeat sequences in either the original (forward span; maximum 12 points) or reverse order (backward span; maximum 10 points) immediately after presentation. Sequence length increases following correct repetition, with testing terminated after two consecutive failures at a given span length. Total scores (sum of both spans) were calculated, with higher scores reflecting better performance.

For the VFT, participants were asked to name as many animals or vegetables as possible within 60 seconds. The average number of items generated across both categories served as the VFT score.

In the AVLT-H [4], a list of 12 words was orally presented three times. Recall was assessed after a 5-minute delay (short-delayed recall) and a 20-minute delay (long-delayed recall).

**REFERENCES**

[1]	Trzepacz PT, Hochstetler H, Wang S, Walker B, Saykin AJ. Relationship between the Montreal Cognitive Assessment and Mini-mental State Examination for assessment of mild cognitive impairment in older adults. BMC Geriatr. 2015;15:107. <https://doi.org/10.1186/s12877-015-0103-3>

[2]	Rosano C, Perera S, Inzitari M, Newman AB, Longstreth WT, Studenski S. Digit Symbol Substitution test and future clinical and subclinical disorders of cognition, mobility and mood in older adults. Age Ageing. 2016;45(5):688-95. <https://doi.org/10.1093/ageing/afw116>

[3]	Yu TC, Wu PT, Wu WL, Chang YK, Chiang CH, Chu IH. Effects of Exergame Training on Executive Function and Heart Rate Variability in Middle-Aged and Older Adults: A Randomized Controlled Study. Eur J Sport Sci. 2025;25(2):e12249. <https://doi.org/10.1002/ejsc.12249>

[4]	Xie XY, Huang LY, Liu D, Cheng GR, Hu FF, Zhou J, et al. Predicting Progression to Dementia Using Auditory Verbal Learning Test in Community-Dwelling Older Adults Based On Machine Learning. Am J Geriatr Psychiatry. 2025;33(5):487-99. <https://doi.org/10.1016/j.jagp.2024.10.016>

**Supplementary Fig. 1** Group comparisons of AD and MD in substantia nigra subregions relative to epileptic focus. **(a-b)** No significant group differences in AD were detected in either SNr or SNc. **(c-d)** No significant group differences in MD were detected in either SNr or SNc. Violin plots show the full data distribution; points represent individual participant values; internal lines indicate the median. AD, axial diffusivity; MD, mean diffusivity; RTLE, right temporal lobe epilepsy; LTLE, left temporal lobe epilepsy; HC, healthy control; SNr, substantia nigra pars reticulata; SNc, substantia nigra pars compacta; Ipsi, ipsilateral; Contra, contralateral

**Supplementary T****able 1** Group × Side interaction effects for SN imaging metrics between RTLE and LTLE with age and sex as covariates

|  |  | **Group × Side interaction** | | | **LTLE** |  |  | **RTLE** |  |  |
| --- | --- | --- | --- | --- | --- | --- | --- | --- | --- | --- |
| **Metric** | **Region** | **F(1,37)** | ***P**** | **partial η²** | **Mean Difference  (ipsi. – contra.)** | ***P**** | **95% CI** | **Mean Difference  (ipsi. – contra.)** | ***P**** | **95% CI** |
| **Susceptibility** | SNr | 0.362 | 0.551 | 0.010 |  |  |  |  |  |  |
|  | SNc | 0.213 | 0.647 | 0.006 |  |  |  |  |  |  |
| **FA** | SNr | 28.526 | **＜0.001** | 0.435 | -0.033 | **＜0.001** | -0.049, -0.018 | 0.025 | **0.003** | 0.009, 0.041 |
|  | SNc | 10.226 | **0.003** | 0.217 | **-0.024** | **0.005** | -0.041, -0.008 | 0.014 | 0.112 | -0.003, 0.031 |
| **RD(*10^-4^)** | SNr | 59.19 | **＜0.001** | 0.615 | 0.316 | **＜0.001** | 0.212, 0.420 | -0.253 | **＜0.001** | -0.360, -0.147 |
|  | SNc | 14.96 | **＜0.001** | 0.288 | 0.204 | **0.008** | 0.058, 0.351 | -0.201 | **0.010** | -0.351, -0.050 |
| **MD(*10^-4^)** | SNr | 10.644 | **0.002** | 0.223 | 0.144 | **0.010** | 0.036, 0.251 | -0.106 | 0.058 | -0.217, 0.004 |
|  | SNc | 1.368 | 0.250 | 0.036 |  |  |  |  |  |  |
| **AD(*10^-3^)** | SNr | 0.032 | 0.859 | 0.001 |  |  |  |  |  |  |
|  | SNc | 1.629 | 0.210 | 0.042 |  |  |  |  |  |  |

**P*-values less than 0.05 were considered significant. SNr, substantia nigra pars reticulata; SNc, substantia nigra pars compacta; FA, fractional anisotropy; RD, radial diffusivity; MD, mean diffusivity; AD, axial diffusivity; η²p, partial eta squared; CI, confidence interval; RTLE, right temporal lobe epilepsy; LTLE, left temporal lobe epilepsy; ipsi., ipsilateral; contra., ontralateral. Mean Difference represents the difference between ipsilateral and contralateral sides (ipsilateral minus contralateral)

**Supplementary Table 2** Group × Side interaction effects for SN imaging metrics between RTLE and LTLE with age, sex, and HS as covariates

|  |  | **Group × Side interaction** | | | **LTLE** |  |  | **RTLE** |  |  |
| --- | --- | --- | --- | --- | --- | --- | --- | --- | --- | --- |
| **Metric** | **Region** | **F(1,37)** | ***P**** | **partial η²** | **Mean Difference (ipsi. – contra.)** | ***P**** | **95% CI** | **Mean Difference (ipsi. – contra.)** | ***P**** | **95% CI** |
| **Susceptibility** | SNr | 0.063 | 0.803 | 0.002 |  |  |  |  |  |  |
|  | SNc | 0.227 | 0.637 | 0.006 |  |  |  |  |  |  |
| **FA** | SNr | 23.382 | **＜0.001** | 0.394 | -0.033 | **＜0.001** | -0.049, -0.017 | 0.025 | **0.005** | 0.008, 0.041 |
|  | SNc | 6.868 | **0.013** | 0.160 | -0.022 | **0.014** | -0.039, -0.005 | 0.011 | 0.205 | -0.006, 0.029 |
| **RD(*10^-4^)** | SNr | 47.472 | **＜0.001** | 0.569 | 0.305 | **＜0.001** | 0.197, 0.412 | -0.241 | **＜0.001** | -0.352, -0.131 |
|  | SNc | 13.527 | **0.001** | 0.273 | 0.209 | **0.009** | 0.056,0.363 | -0.206 | **0.012** | -0.364, -0.049 |
| **MD(*10^-4^)** | SNr | 9.18 | **0.005** | 0.203 | 0.144 | **0.014** | 0.032, 0.257 | 0.107 | 0.069 | -0.009, 0.223 |
|  | SNc | 1.83 | 0.185 | 0.048 |  |  |  |  |  |  |
| **AD(*10^-3^)** | SNr | 0.002 | 0.966 | 0 |  |  |  |  |  |  |
|  | SNc | 0.678 | 0.416 | 0.018 |  |  |  |  |  |  |

**P*-values less than 0.05 were considered significant. SNr, substantia nigra pars reticulata; SNc, substantia nigra pars compacta; FA, fractional anisotropy; RD, radial diffusivity; MD, mean diffusivity; AD, axial diffusivity; η²p, partial eta squared; CI, confidence interval; RTLE, right temporal lobe epilepsy; LTLE, left temporal lobe epilepsy; HS, hippocampal sclerosis; ipsi., ipsilateral; contra., ontralateral. Mean Difference represents the difference between ipsilateral and contralateral sides (ipsilateral minus contralateral)
